# r84, a Novel Therapeutic Antibody against Mouse and Human VEGF with Potent Anti-Tumor Activity and Limited Toxicity Induction

**DOI:** 10.1371/journal.pone.0012031

**Published:** 2010-08-06

**Authors:** Laura A. Sullivan, Juliet G. Carbon, Christina L. Roland, Jason E. Toombs, Mari Nyquist-Andersen, Anita Kavlie, Kyle Schlunegger, James A. Richardson, Rolf A. Brekken

**Affiliations:** 1 Division of Surgical Oncology, Department of Surgery, Hamon Center for Therapeutic Oncology Research, University of Texas Southwestern Medical Center, Dallas, Texas, United States of America; 2 Affitech AS, Oslo Research Park, Oslo, Norway; 3 Peregrine Pharmaceuticals, Inc., Tustin, California, United States of America; 4 Departments of Pathology and Molecular Biology, University of Texas Southwestern Medical Center, Dallas, Texas, United States of America; 5 Department of Pharmacology, University of Texas Southwestern Medical Center, Dallas, Texas, United States of America; University of Illinois at Chicago, United States of America

## Abstract

Vascular endothelial growth factor (VEGF) is critical for physiological and pathological angiogenesis. Within the tumor microenvironment, VEGF functions as an endothelial cell survival factor, permeability factor, mitogen, and chemotactic agent. The majority of these functions are mediated by VEGF-induced activation of VEGF receptor 2 (VEGFR2), a high affinity receptor tyrosine kinase expressed by endothelial cells and other cell types in the tumor microenvironment. VEGF can also ligate other cell surface receptors including VEGFR1 and neuropilin-1 and -2. However, the importance of VEGF-induced activation of these receptors in tumorigenesis is still unclear. We report the development and characterization of r84, a fully human monoclonal antibody that binds human and mouse VEGF and selectively blocks VEGF from interacting with VEGFR2 but does not interfere with VEGF∶VEGFR1 interaction. Selective blockade of VEGF binding to VEGFR2 by r84 is shown through ELISA, receptor binding assays, receptor activation assays, and cell-based functional assays. Furthermore, we show that r84 has potent anti-tumor activity and does not alter tissue histology or blood and urine chemistry after chronic high dose therapy in mice. In addition, chronic r84 therapy does not induce elevated blood pressure levels in some models. The ability of r84 to specifically block VEGF∶VEGFR2 binding provides a valuable tool for the characterization of VEGF receptor pathway activation during tumor progression and highlights the utility and safety of selective blockade of VEGF-induced VEGFR2 signaling in tumors.

## Introduction

Angiogenesis is a tightly regulated process that is essential during growth, wound healing and development, as well as cancer growth, progression and metastasis [1]–[2]. A key stimulant of angiogenesis is vascular endothelial growth factor-A (VEGF). VEGF induces endothelial cell survival, proliferation, and migration its predominant signaling receptor, VEGF receptor 2 (VEGFR2). Tumor associated macrophages also express VEGFR2 and selective blockade of VEGFR2 is able to decrease macrophage infiltration into tumors [3]. VEGF signaling through VEGF receptor 1 (VEGFR1) remains unclear, although it is thought to have effects on hematopoiesis, vascular permeability, and monocyte migration. Importantly, there is elevated expression of VEGF, VEGFR1, and VEGFR2 within tumors, providing a therapeutic target. In fact targeting VEGF has lead to the development of anti-angiogenic therapies such as sunitinib malate (Sutent®, SU11248, Pfizer, Inc.), sorafenib (Nexavar®, BAY 43-9006, Bayer Pharmaceuticals Corp.), bevacizumab (Avastin®, Genentech), IMC-1121b (ramucirumab, ImClone), VEGF-Trap (aflibercept, Regeneron) and 2C3 [2], [4]–[6].

Sunitinib and sorafenib are small molecule inhibitors of multiple receptor tyrosine kinases (RTKs) including the VEGF receptors. These drugs have been FDA-approved for the treatment of renal cell carcinoma, gastrointestinal stromal tumors (GIST) (sunitinib), and unresectable hepatocellular carcinoma (sorafenib) [2], [7]–[8].

Bevacizumab is a humanized monoclonal anti-VEGF antibody that inhibits VEGF from binding to and signaling through VEGFR1 and VEGFR2. Bevacizumab is approved in combination with cytotoxic chemotherapy for the treatment of colorectal cancer, non-small cell lung cancer (NSCLC), and breast cancer, as monotherapy for glioblastoma, and in combination with interferon for renal cell carcinoma [9]–[11]. Treatment with bevacizumab in these cancer types results in a delay of tumor progression and increases in patient survival [2], [9]. However, treatment with bevacizumab, sorafenib, and sunitinib, is also associated with a number of rare although serious toxicities including gastro-intestinal perforations, bleeding, proteinurea, and glomerulosclerosis [9], [12]–[13].

IMC-1121b is a high affinity, fully human IgG1 monoclonal antibody that recognizes VEGFR2. IMC-1121b binding to VEGFR2 inhibits ligand-induced activation of the receptor. There are several on-going phase I, II, and III clinical trials evaluating the efficacy of IMC-1121b in a number of tumor types [6].

VEGF-Trap is comprised of the second and third extracellular immunoglobulin domains of VEGFR1 and VEGFR2, respectively, joined by an IgG1 Fc region. The resulting fusion protein traps with high affinity multiple VEGF family members including VEGF and placental growth factor (PlGF) [14]. Currently, VEGF-Trap is being tested in phase III clinical trials in a number of tumor types [6].

2C3 is a murine, monoclonal antibody against VEGF that specifically blocks human VEGF binding to VEGFR2 [4]. The selective inhibition of VEGF∶VEGFR2 signaling by 2C3 reduces vascular permeability, decreases endothelial cell growth, and decreases tumor growth in murine xenograft models. Additionally, 2C3 reduces tumor microvessel density (MVD) and macrophage infiltration and down-regulates VEGFR2 expression on the tumor vasculature [3]–[5], [15]. The desirable anti-angiogenic effects of 2C3 lead to the development of a human antibody that retains 2C3 specificity.

Here we describe a fully human monoclonal antibody, r84 (AT001, Affitech AS) that binds to mouse and human VEGF and specifically inhibits VEGF binding to VEGFR2, while leaving intact VEGF interaction with VEGFR1. Through blockade of VEGFR2 signaling, r84 inhibits the migration of VEGFR2 positive endothelial cells, and blocks VEGFR2 phosphorylation and downstream signaling. In addition, treatment of mice bearing tumor xenografts with r84 delays tumor take resulting in tumor vascular changes, including reductions in tumor MVD and in tumor lymphatic vessel density (LVD). Furthermore, extended treatment with r84 does not induce significant systemic toxicity in mice.

## Materials and Methods

### Construction of human IgG anti-VEGF antibodies

Human anti-VEGF single chain variable fragments (scFvs) were created by Affitech AS (Olso, Norway) and Peregrine Pharmaceuticals, Inc. (Tustin, CA) and screened for specific VEGF binding characteristics. The most desirable scFvs were cloned into full length antibody expression vectors containing the glutamine synthetase gene, transfected into CHO K1SV cells, and selected in a glutamine free cell culture media. The cells were plated into flat bottom 96 well culture plates, and wells with antibody production were diluted and the cells were subcloned. Once subcloned, the high production cells were grown to 500 mL cultures and the antibody was purified by Protein-A affinity chromatography and size-exclusion chromatography for purities of greater than 90% monomer.

### ELISA analysis of r84

To evaluate the binding specificities of r84, a series of ELISAs were performed.

#### Determination of r84 specificity

Relative binding affinity of r84 for mouse and human VEGF was determined by ELISA. Recombinant human VEGF (R&D Systems®, Minneapolis, MN) or mouse VEGF (Sigma-Aldrich®, St. Louis, MO) was coated onto the bottom of 96-well plates at 0.5 µg/mL. Wells were blocked and then incubated with r84 starting at 2 µg/mL with a serial dilution factor of four. Antibody bound to the wells was detected by incubation with anti-human Fc HRP-conjugated antibody followed by development with HRP substrate.

#### r84 specificity within VEGF family

Human VEGF-A, mouse VEGF-A, human VEGF-B, human VEGF-C, human VEGF-D, and human PlGF (R&D Systems®) were coated onto 96-well ELISA plates at 0.5 µg/mL. Wells were blocked and then incubated with human r84 at 1 µg/mL. Antibody bound to the wells was detected as described above.

#### r84 receptor blocking ELISAs

Recombinant human VEGFR1/Fc or VEGFR2/Fc (R&D Systems®) was coated onto the bottom of 96-well plates at 1 µg/mL. Wells were blocked and then incubated with 2.38 nM or 4.76 nM biotinylated VEGF for VEGFR1 and VEGFR2, respectively, +/− fold the indicated molar excess of antibody. Labelled VEGF bound to the wells was detected by incubation with strepavidin HRP-conjugated antibody, developed as described above, and displayed as a percentage of VEGF binding alone in the absence of antibody.

### Endothelial cell *in vitro* assays

The effect of r84 on endothelial cell function and signaling *in vitro* was assessed using HDMEC (ScienCell™ Research Laboratories, Carlsbad, CA), PAE-KDR [16], PAE-Flt-1 [16] endothelial cell lines.

#### Migration assays

A modified Boyden chamber assay was used. 20,000 endothelial cells (ECs) (HDMEC (ScienCell™ Research Laboratories, Carlsbad, CA), PAE-KDR [16], PAE-Flt-1 [16]) were plated in serum free media on 8.0 µm pore size cell culture inserts (BD Falcon™, San Jose, CA) and allowed to migrate overnight at 37°C. Recombinant human or mouse VEGF (Sigma-Aldrich®) was used as a chemo-attractant at 100 ng/mL, with antibodies added at a 500-fold molar excess. Insert membranes were isolated following migration and stained with DAPI to allow for quantification of migrated cells (total magnification, 100×).

#### Stimulation assays

HDMEC and PAE-KDR, -Flt-1 cell lines were maintained in 100 mm^2^ tissue culture dishes in MCDB 131 (Gibco®, Carlsbad, CA) media supplemented with 0.4 µg/mL ECGF and 20% fetal bovine serum (FBS). Following 24 hour serum starvation, cells were stimulated for two minutes with 100 ng recombinant human VEGF or mouse (R&D Systems®) +/− 500-fold molar excess antibody. Cell lysates of stimulated cells were prepared and analyzed by Western blot using commercially-available antibodies specific for targets of interest ( total and phospho- VEGFR2, p38, PLCγ, Erk1/2 (Cell Signaling Technology®, Danvers, MA), and VEGFR1 (Abcam®, Cambridge, MA)).

### Animal studies

4–6-week-old NOD/SCID mice were purchased from the breeding core at the University of Texas Southwestern Medical Center. Animals were housed in a pathogen-free facility and all procedures were performed in accordance with a protocol (APN 0974-07-05-1) approved by the IACUC of the University of Texas Southwestern Medical Center.

#### Tumor models and treatment

All tumor cells (H460, H1299, A549, PANC-1) were grown in culture in RPMI-1640 medium (HyClone®, Waltham, MA) supplemented with 5% FBS. Cell lines were confirmed to be pathogen free and were authenticated to confirm origin prior to use.

#### Subcutaneous xenograft therapy study

2.5 million H460, H1299, A549 NSCLC cells (provided by Dr. John Minna) were injected (in PBS) subcutaneously into the right flank of NOD/SCID mice. Mice were treated with 50 mg/kg/week r84 and 25 mg/kg/week bevacizumab/Avastin® and palivizumab/Synagis® (anti-respiratory syncytial virus) via intraperitoneal (IP) injection starting one day post tumor cell injection (TCI) (n = 8–9/group). Mice were monitored twice a week, recording weights, taking perpendicular tumor measurements, and observing for signs of distress such as weight loss and inactivity. Therapy continued until average control-treated tumor volume reached 1500 mm^3^ or until day 60 post TCI, at which point animals were sacrificed.

#### Toxicity studies

5 million PANC-1 human pancreatic cancer cells (ATCC, Manassas, VA) (in PBS) were injected subcutaneously into the right flank of NOD/SCID mice. An equal number of NOD/SCID mice were not injected with tumor cells. Therapy began one day post TCI. Tumor bearing (TB) and non-tumor bearing (NTB) mice were treated with 50 mg/kg/week r84 and palivizumab via IP injection. Each group consisted of five mice. Mice were monitored as previously described. All mice were sacrificed following 12 weeks of continuous therapy and evaluated for r84-induced toxicity. Blood was collected from animals at sacrifice; serum was isolated following centrifugation and analyzed by the mouse metabolic phenotyping core at the University of Texas Southwestern Medical Center. A second toxicity study was performed in immunocompetent mice harbouring spontaneous pancreatic cancer (*p48cre/Kras^G12D^/INK4a*) [17]. Mice were treated with saline (n = 4) or 25 mg/kg/week mouse chimeric r84 (mcr84, n = 3) via IP injection or with 50 mg/kg/week sunitinib (n = 4) by daily oral gavage five days per week. Sunitinib was purchased from LC laboratories (Woburn, MA). Therapy began when mice reached eight weeks old. Mice were monitored for weight gain as previously described. At weeks two and seven of therapy, tail vein cuff blood pressures of all mice were measured using the Visitech Systems BP-2000 Series II Blood Pressure Analysis System™ through the O'Brien Kidney Research Core Center at the University of Texas Southwestern Medical Center. To familiarize mice to the procedure, tail cuff blood pressures were measured for five consecutive days, with data collection on the fifth day. Average systolic pressures were calculated from data collected on the last day of measurement (day five). At week six of therapy metabolic cages obtained through the O'Brien Kidney Research Core Center at the University of Texas Southwestern Medical Center were used to collect urine from all animals over a 24-hour collection period. Fresh urine samples were then submitted to the University of Texas Southwestern Medical Center mouse metabolic phenotyping core for analysis of total levels of urine protein and creatine. All mice were sacrificed following eight weeks of continuous therapy and evaluated for mcr84- and sunitinib-induced toxicity. Blood was collected from animals at sacrifice; serum was isolated following centrifugation and analyzed by the mouse metabolic phenotyping core at the University of Texas Southwestern Medical Center.

#### Therapy dose titration

2.5 million A549 NSCLC cells (in PBS) were injected subcutaneously into the right flank of NOD/SCID mice. Mice were treated with 5, 15, or 50 mg/kg/week r84 and bevacizumab and 15 mg/kg/week control IgG (Peregrine Pharmaceuticals, Inc.) via IP injection starting one day post tumor cell injection (TCI). Each group consisted of eight mice and were monitored as above. Therapy continued until average control-treated tumor volume reached 1200 mm^3^, at which point animals were sacrificed.

### Histology and Immunohistochemical Studies

Formalin-fixed, paraffin-embedded tissues were sectioned and stained with hematoxylin and eosin by the molecular pathology core laboratory at the University of Texas Southwestern Medical Center. Snap frozen tumors were sectioned, blocked with 20% Aquablock (East Coast Biologics, North Berwick, ME) and stained for markers of interest. Primary antibodies used include MECA-32 (DSHB; University of Iowa), endomucin (Santa Cruz Biotechnology®, Inc., Santa Cruz, CA), NG2 (Millipore®, Billerica, MA), smooth muscle actin (NeoMarkers, Fremont, CA), Lyve1, VEGFR2 (55B11) (Cell Signaling Technology®, Danvers, MA), rabbit anti-VEGFR2 T014 (purified in our laboratory) [4], [18], rat anti-VEGFR2 RAFL-2 [19], and insulin (Dako, Glostrup, Denmark).

### Statistics

Data were analyzed using GraphPad software (GraphPad Prism version 5.00 for Windows, GraphPad Software). Results are expressed as mean ± SE. Differences are analyzed by *t* test or ANOVA, and results are considered significant at a *p* value of <0.05.

## Results

### Generation of a fully human monoclonal antibody against VEGF

The success of 2C3 in preclinical models led to the development of a fully human monoclonal antibody that recognizes VEGF and retains many of the characteristics of 2C3. A number of anti-VEGF human single chain variable fragments (scFv) were screened for several characteristics such as a competition with 2C3 for binding to VEGF, the ability to block VEGF∶VEGFR2 binding, and the ability of the scFv to bind to different VEGF isoforms such as VEGF165 and VEGF121.

### r84 binds human and mouse VEGF-A and specifically blocks VEGF from binding to VEGFR2

**Figure 1:**
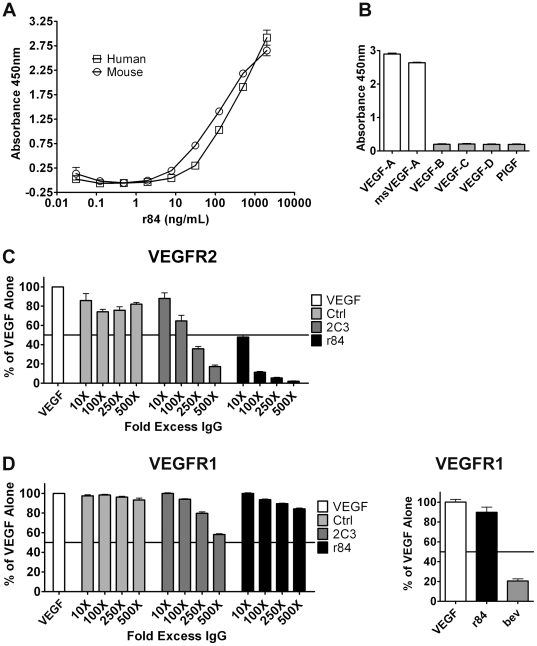
r84 binds human and mouse VEGF-A and specifically blocks VEGF-A binding to VEGFR2, not VEGFR1. *A*, Recombinant human VEGF coated at 0.5 µg/mL was detected with a titration of fully human monoclonal antibody r84. r84 bound to VEGF was detected with an anti-human Fc HRP-conjugated antibody, demonstrating r84 binds both human and mouse VEGF-A (open squares and circles, respectively). *B*, Recombinant human and mouse VEGF-A, and human VEGF-B, -C, -D, and PlGF coated at 0.5 µg/mL was detected with r84 at 1 µg/mL. Binding of r84 to VEGF family member was detected as in *A*, demonstrating r84 binds only human and mouse VEGF-A and not other VEGF family members. VEGFR2 (*C*) and VEGFR1 (*D*, *left panel*) coated at 1 µg/mL were incubated with 4.76 nM or 2.38 nM biotinylated VEGF, respectively, +/− the indicated fold excesses of antibody (Control IgG, 2C3, r84). r84, 2C3 specifically block biotinylated-VEGF binding to VEGFR2 (*C*), but not VEGFR1 (*D*, *left panel*). In contrast, a 500-fold molar excess bevacizumab (bev) reduces biotinylated-VEGF binding to VEGFR1, compared to biotinylated-VEGF alone or plus r84 (*D*, *right panel*).

To determine the binding specificity of r84, a series of ELISAs were performed. A titration of r84 against recombinant human or mouse VEGF demonstrated that r84 binds with equal affinity to both species (Figure 1A). This established r84 as an important tool in evaluating the contribution of both tumor cell- and host-derived VEGF in tumor progression using xenograft models. The binding specificity of r84 differs from other anti-VEGF antibodies, such as bevacizumab and 2C3 that recognize only human VEGF. Next, the specificity of r84 within the VEGF family was determined. r84 only bound wells coated with recombinant human and mouse VEGF-A (Figure 1B).

The effect of r84 on VEGF binding to VEGFR1 and VEGFR2 was determined using ligand-receptor ELISAs. 2C3 and r84 at increasing fold molar excess significantly reduced biotinylated-VEGF binding to VEGFR2, compared to binding of biotinylated-VEGF alone or in the presence of a non-specific control IgG (Figure 1C). In contrast, neither 2C3 nor r84 inhibited binding of biotinylated-VEGF to VEGFR1 (Figure 1D, left panel). However, at a 500 fold molar excess of antibody to biotinylated-VEGF, bevacizumab decreased VEGF binding to VEGFR1 by approximately 80% (Figure 1D, right panel). These blocking ELISAs demonstrate the precise binding of r84 to VEGF to selectively inhibit the VEGF∶VEGFR2 interaction.

### r84 effects VEGFR2-mediated endothelial cell function

**Figure 2:**
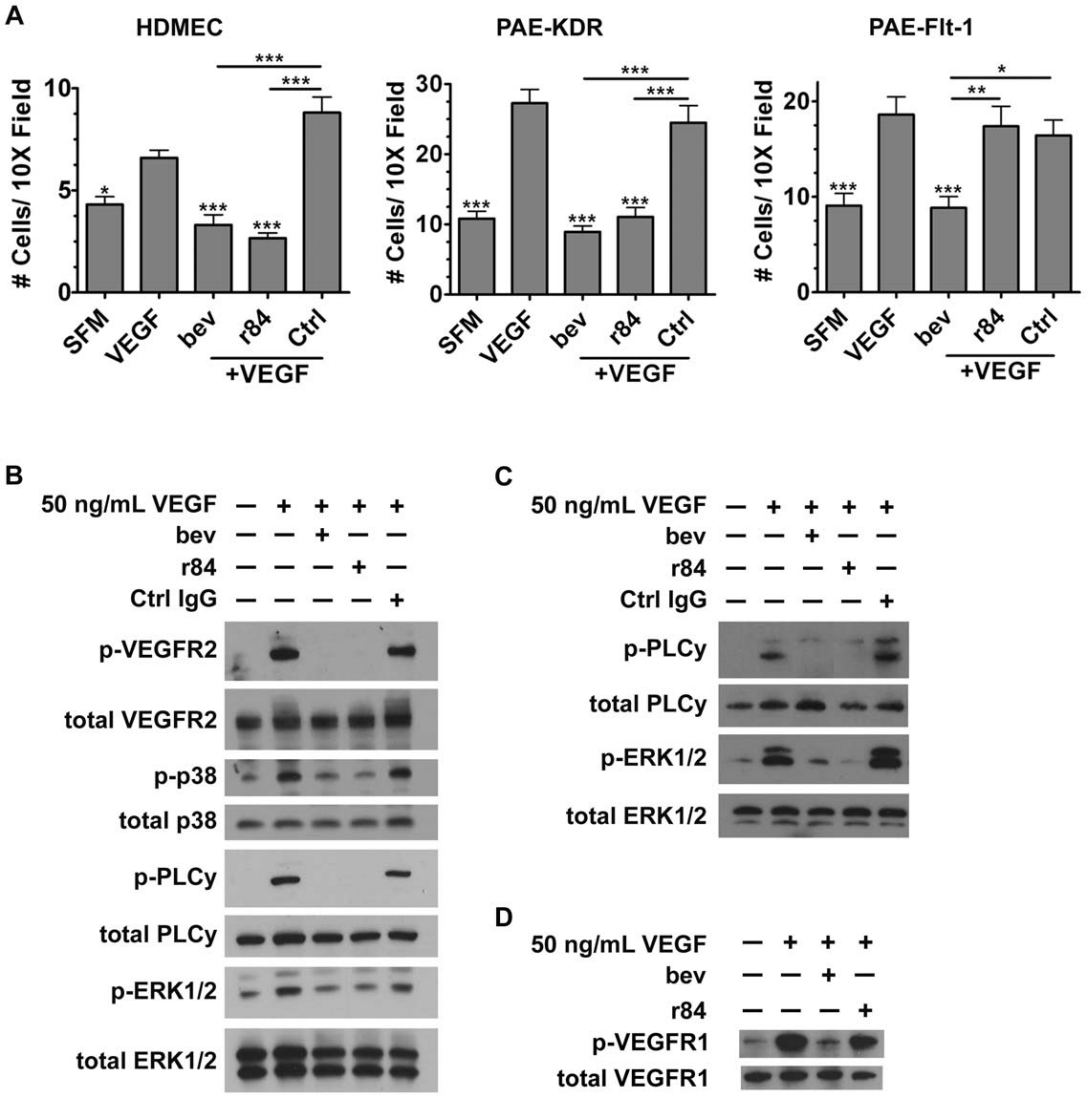
r84 reduces endothelial cell migration and signaling *in vitro*. *A*, A modified Boyden chamber migration assay was used to assess the effect of r84, bevacizumab (bev) on VEGF-induced endothelial cell (EC) migration. 20,000 HDMEC, PAE-KDR, PAE-Flt-1 cells were plated on 8.0 µm cell culture inserts and allowed to migrate overnight towards SFM or human VEGF (100 ng/mL) +/− 500-fold molar excess antibody (bev, r84, control IgG). r84, bev block the VEGF-induced migration of VEGFR2-expressing ECs (*A*, *HDMEC*, *PAE-KDR*). Bev blocks VEGF-induced migration of endothelial cells expressing VEGR1, but r84 does not (*A, PAE-Flt-1*). Western blots of VEGF-induced signaling in PAE-KDR (*B*), HDMEC (*C*), and PAE-Flt-1 (*D*) lysates following stimulation of cells with 50 ng/mL human VEGF +/− 500-fold molar excess antibody (bev, r84, control IgG). r84 and bev block p-VEGFR2 and downstream phosphorylation (p38, PLCγ, ERK1/2) (*B*, *C*), but only bev blocks VEGF-induced VEGFR1 phosphorylation in PAE-Flt-1 stimulated cells (*D*). *p<0.05, **p<0.01, ***p<0.001, statistical differences in *A* compared to VEGF alone, unless otherwise indicated.

The effect of r84 on endothelial cells was determined using several *in vitro* assays. First, a transwell assay was used to test the effects of anti-VEGF antibodies on VEGF-induced endothelial cell migration. Three different endothelial cell lines, selected for their VEGF receptor expression, were used for the migration assays. Human dermal microvascular endothelial cells (HDMEC) express VEGFR1 and VEGFR2, porcine aortic endothelial cells (PAE)-KDR and PAE-Flt-1 express high levels of VEGFR2 or VEGFR1, respectively [16]. Human VEGF significantly induced migration of all three cell types compared to serum free media (SFM) alone (p<0.05 for HDMEC, p<0.001 for PAE-KDR, -Flt-1), and a non-specific control IgG did not affect VEGF-induced migration (Figure 2A). Both r84 and bevacizumab significantly inhibited VEGF-induced migration of VEGFR2-expressing endothelial cells (p<0.001, Figure 2A, HDMEC, PAE-KDR). However, only bevacizumab was able to decrease the migration of PAE-Flt-1 cells towards VEGF (Figure 2A, PAE-Flt-1). To further evaluate the specificity of r84 to mouse VEGF, migration assays were performed with PAE-KDR cells using mouse VEGF as the chemotactic agent. As seen with human VEGF, mouse VEGF significantly induced the migration of PAE-KDR cells as compared to SFM alone (Figure S1A). Only r84 was able to significantly inhibit this migration, while bevacizumab and a control IgG had no effect on cell migration (p<0.001, Figure S1A). The ability of r84 to specifically block both human and mouse VEGF-induced migration of VEGFR2-expressing endothelial cells (HDMEC, PAE-KDR) but not VEGFR1-expressing endothelial cells (PAE-Flt-1) demonstrates the selectivity of r84 to inhibit VEGF-induced VEGFR2 activity.

VEGF binding to VEGFR2 initiates receptor phosphorylation and subsequent phosphorylation of downstream pathway components such as phospholipase C gamma (PLCγ), p38, and the MAP kinase extracellular signal-regulated kinase (ERK1/2). PAE-KDR cells stimulated *in vitro* with human VEGF (100 ng, two minutes) induced phosphorylation of VEGFR2, PLCy, p38, and ERK (Figure 2B). Human VEGF stimulation of HDMECs induced phosphorylation of PLCγ and ERK (Figure 2C). Stimulation of PAE-KDR and HDMEC cells with human VEGF plus 500-fold molar excesses r84 or bevacizumab inhibited phosphorylation of VEGFR2 and downstream targets (Figure 2B, 2C). However, only bevacizumab blocked human VEGF-induced phosphorylation of VEGFR1 in PAE-Flt-1 (Figure 2D). Further, stimulation of PAE-KDR cells with mouse VEGF induced phosphorylation of VEGF2, PLCy, and ERK that was only inhibited by r84 and not by bevacizumab or a control IgG (Figure S1B). This data shows that r84 selectively inhibits human and mouse VEGF binding and signaling through VEGFR2 without interrupting VEGFR1 signaling. The ability of r84 to bind human and mouse VEGF (Figure 1A, B) and block VEGF from binding and signaling through VEGFR2 (Figure 1C, Figure 2A–D, Figure S1A, B) makes r84 a unique tool for studying VEGF inhibition in tumor xenograft models, assessing possible toxicity induction and analyzing the importance of VEGFR1 signaling in these processes.

### r84 delays take of human xenograft tumors

**Figure 3:**
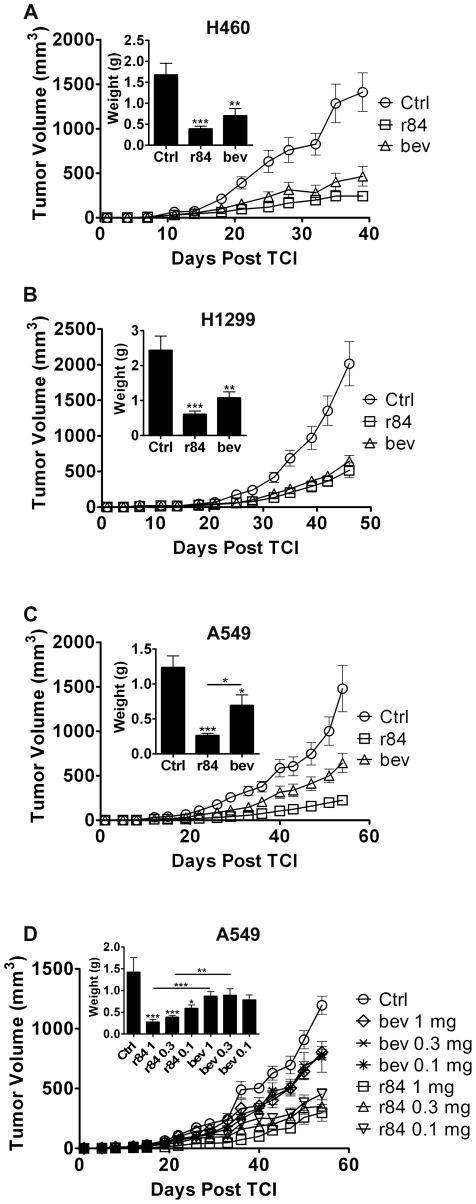
r84 controls tumor growth *in vivo*. 2.5 million human NSCLC cells were injected subcutaneously into the right flank of NOD/SCID mice. Therapy began one day post tumor cell injection (TCI), and continued for 4–8 weeks. Tumor volumes were measured twice/week and final tumor weights were recorded at sacrifice. r84 and bevacizumab (bev) similarly control tumor growth and final weight compared to control IgG treatment in H460, H1299 models (*A*, *B*). *C*, In A549 NSCLC tumor bearing animals, r84's control of tumor growth was significantly different from bev (p<0.05). *D*, Titration of antibody dosing in A549 tumor xenografts showed no change in tumor growth and final tumor weight with increasing doses of bev, however there was increasing control of tumor growth with increasing doses of r84, with doses of r84 controlling growth better than bev (*D*). *A–C*, n = 8 mice per group; *D*, n = 6 mice per group. *p<0.05, **p<0.01, ***p<0.001, statistical differences compared to Ctrl treatment, unless otherwise indicated.

Previous studies in our lab have demonstrated the ability of r84 to control tumor growth and decrease tumor angiogenesis in established models of breast cancer [20]–[21]. The efficacy of r84 as a cancer therapeutic was further assessed in tumor xenograft models in NOD/SCID mice. Briefly, four- to six-week old female NOD/SCID mice were implanted subcutaneously (s.c.) with 2.5 million human non-small cell lung cancer (NSCLC) cell lines H460, H1299, or A549. Treatment began one day post tumor cell injection (TCI) and continued until the average tumor volume in control IgG treated tumors reached 1500 mm^3^, at which time all animals were sacrificed. Tumor-bearing animals were treated with 50 mg/kg/week r84 and 25 mg/kg/week bevacizumab and control IgG (palivizumab/Synagis®). r84 and bevacizumab similarly delayed tumor take, thereby controlling H460 and H1299 tumor growth compared to control IgG therapy by both tumor volume and final tumor weights at sacrifice (Figure 3A, B). In A549 xenografts, r84 delayed tumor take better than bevacizumab, and the mean final tumor weight at sacrifice of animals treated with r84 was significantly smaller than animals treated with bevacizumab (Figure 3C, p<0.05).

Bevacizumab has a half life in mice of approximately two weeks [22]. Pharmacokinetic studies (data not shown) determined the half life of r84 in mice to be approximately five days. This difference, along with the fact that r84 binds both human and mouse VEGF and thus has more target to bind in tumor xenograft models than bevacizumab, led to the differences in antibody doses used in tumor studies. Consequently, this increase in dose could lead to better control of tumor growth as was seen in the A459 model (Figure 3C). To evaluate the effect of antibody dose, A549 tumor cells were implanted into mice as previously described. One day post TCI, animals began therapy, receiving 5, 15, or 50 mg/kg/week of r84 or bevacizumab, or 15 mg/kg/week of a non-specific control human IgG. The different doses of bevacizumab had the same effect on tumor growth and final tumor weight (Figure 3D). In contrast, there was an observable titration of tumor growth and final tumor weight with r84 therapy, with tighter control seen at higher doses of antibody. In addition, treatment of A549 tumor-bearing animals with 15 and 50 mg/kg/week r84 resulted in smaller tumors as compared to the same dose of bevacizumab (Figure 3D, p<0.001 and p<0.01, respectively). Therefore, these results indicate that r84 may be more effective at controlling tumor take and growth than bevacizumab independent of dose in certain models. We propose that the appropriate therapeutic antibody dose should be determined independently for different tumor types to maximize therapeutic benefit with minimal induction of toxicity [23]–[25].

### r84 effects the tumor microenvironment

**Figure 4:**
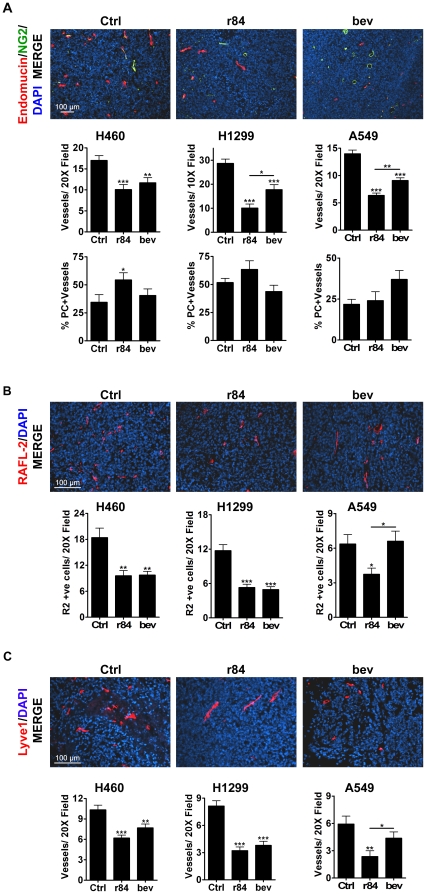
r84 therapy induces vascular changes within tumors. Frozen sections of A549, H460, H1299 tumors treated with control IgG (Ctrl), r84, or bevacizumab (bev) were analyzed by immunofluorescence for endothelial, pericyte (PC), and lymphatic markers, as well as for VEGFR2 expression. Number of positive-staining entities per high powered field was evaluated using Nikon Elements software. *A*, r84, bev treatment significantly decreases tumor MVD, shown by a reduction in tumor endomucin positive endothelial cells (red). r84, bev treatment induced a trend towards increased NG2 positive (green) PC coverage of vessels as compared to Ctrl. *B*, r84, bev treatment significantly reduces the number of VEGFR2 positive cells in H460, H1299 tumors as shown by RAFL-2 staining (red). Only r84 treatment significantly reduced VEGFR2 staining in A549 tumors. *C*, r84, bev treatment significantly decreased H460, H1299 tumor LVD, as indicated by a reduction in lyve1 positive cells (red). Only r84 treatment significantly reduced A549 tumor LVD. *p<0.05, **p<0.01, ***p<0.001, statistical differences compared to Ctrl, unless otherwise indicated.

The phenotypic effects of r84 therapy within NSCLC tumors were assessed by immunohistochemistry (IHC). As was expected for anti-angiogenic therapies, treatment with r84 and bevacizumab resulted in a significant decrease in tumor MVD as demonstrated using two endothelial cell markers, MECA-32 (data not shown) and endomucin (Figure 4A). There was a trend towards an increase in the number of pericyte-(PC) associated blood vessels in r84- and bevacizumab-treated tumors as compared to control IgG, although this increase only reached significance in the H460 model (Figure 4A). Treatment of H460 and H1299 xenograft tumors with r84 or bevacizumab also reduced the number of VEGFR2 positive cells, as analyzed by IHC (Figure 4B). Interestingly, VEGFR2 expression in A549 tumors was only decreased following r84 and not bevacizumab (Figure 4B, p<0.05) therapy, perhaps reflecting the difference in the efficacy of these two drugs in controlling tumor growth in this model. Additionally, inhibition of VEGF with r84 or bevacizumab decreased tumor LVD as compared to control IgG therapy in both H460 and H1299 models (Figure 4C). However, bevacizumab therapy failed to reduce LVD in the A549 model. These results suggest that in a model-dependent manner, r84 and bevacizumab may be able to disrupt lymphatic vessel-mediated tumor metastasis.

### Extended r84 therapy does not induce toxicity

**Figure 5:**
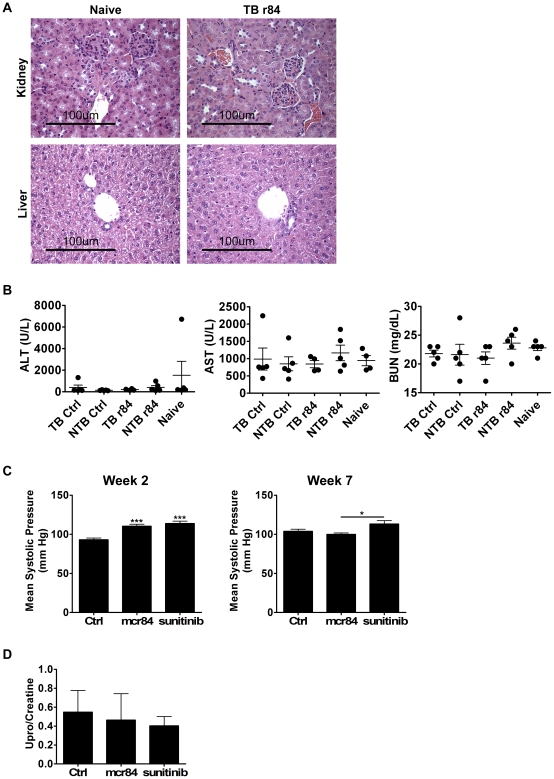
Extended r84 therapy controls tumor growth without induction of toxicity. 5 million PANC-1 tumor cells were injected subcutaneously into NOD/SCID mice. Tumor bearing (TB) and non tumor bearing (NTB) mice received long-term 12-week therapy with 50 mg/kg/week r84 or a control IgG. Following 12-weeks of therapy, animals were sacrificed and organs and blood were collected for toxicity analysis. n = 5 animals per group. *A*, Hematoxylin and eosin staining of formalin-fixed, paraffin-embedded kidney and liver sections demonstrated that control of tumor growth is achieved without induction of kidney or liver histopathologic changes. *B*, Blood chemistry analysis of serum samples collected from mice at sacrifice indicated that r84 treatment does not induce changes in serum levels of alanine aminotransferase (ALT), aspartate aminotransferase (AST), or blood urea nitrogen (BUN) as compared to control IgG therapy and to age-matched Naïve animals that did not have tumor and never received antibody therapy. Immunocompetent Kras/INK4a mice that spontaneously develop pancreatic cancer were treated for eight weeks with saline, 25 mg/kg/week mcr84, or 50 mg/kg/week sunitinib. After 2 weeks of therapy, mcr84 and sunitinib significantly increased mean systolic blood pressure (*C*, left panel, ***p<0.001), but this effect was lost by week 7 of continuous therapy (*C*, right panel). *D*, Urine samples collected during week 6 of therapy and assayed for total levels of urine protein and creatine (Upro/Creatine ratio displayed) showed no significant difference between treated animals as compared to control, indicating that extended therapy with mcr84 and sunitinib did not induce kidney damage in this model.

The use of bevacizumab and other anti-angiogenic therapies in the clinic is associated with a number of rare although serious toxicities. Toxicity associated with r84 can be evaluated in preclinical mouse xenograft models because of its ability of r84 to bind both human and mouse VEGF. To assess the potential of r84 to induce toxicities, NOD/SCID mice were injected with five million PANC-1 tumor cells (a slow-growing human pancreatic cancer line) s.c. Treatment began one day post TCI, with 50 mg/kg/week r84 or a non-specific control IgG (palivizumab/Synagis®). An equal number of non-tumor-bearing (NTB) animals received antibody treatment as well. Therapy continued for 12 weeks, at which point animals were sacrificed and tumor, organs and blood were collected for toxicity assessment. As was seen in the NSCLC models, r84 therapy significantly reduced PANC-1 tumor growth and final tumor weight, as compared to control (Figure S2A, p<0.05). In addition, r84 treatment resulted in decreased tumor MVD (Figure S2B, p<0.001). r84 did not induce histological changes (as assessed by a pathologist) within the kidney or liver of tumor-bearing (TB) or NTB mice as compared to age-matched naïve animals (Figure 5A, naïve and TB r84 displayed). Blood was collected from all animals at the time of sacrifice, and a serum analysis of 20 metabolic markers was performed at UT Southwestern Medical Center's mouse metabolic phenotyping core (Table S1). There were no significant changes in any of these analytes between treated animals and age-matched naïve animals. This analysis included no observable change in alanine aminotransferase, aspartate aminotransferase, and blood urea nitrogen levels (Figure 5B), markers of liver and kidney function, respectively. These three markers are elevated in correlation with toxicity in animals treated for 12 weeks with bevacizumab and high-affinity anti-VEGF antibodies [12].

It has been reported that anti-VEGF treatment can reduce pancreatic islet vascular density in adult mice, leaving the supporting pericytes behind [9]. In this study, the pancreatic islets of TB and NTB animals treated for 12 weeks with r84 showed a reduction in MVD as compared to control IgG-treated TB and NTB animals (p<0.01), but there was no significant change when compared to naïve animals (Figure S3A). Additionally, there was no observable change in the percentage of pericytes without endothelial cell association (Figure S3A). Furthermore, there was no change in serum glucose levels, nor was there a change in insulin staining in pancreatic islets of experimental animals (Figure S3B, C). Taken together, long-term therapy with r84 produced no observable toxicity in TB or NTB animals.

Since hypertension and proteinuria are among the most common toxicity-related side effects associated with anti-VEGF therapy and given recent data suggesting a role for VEGFR2 in controlling blood pressure [13], [26], we investigated the effects of r84 therapy on hypertension and proteinuria in a spontaneous, immunocompetent model of pancreatic cancer. Mice (*p48-Cre:LSL-Kras^G12D^:p16^ink4a/arf+/lox^*) expressing a pancreas-specific Cre recombinanse activating a constitutively active *Kras* allele (*Kras^G12D^*) and inactivating a single copy of *Ink4a/Arf* that spontaneously develop pancreatic ductal adenocarcinoma (PDAC) [17] were separated into three groups receiving either saline, mouse chimeric r84 (mcr84 [21]), or sunitinib. Therapy began when mice when mice were eight weeks old, with weekly IP injections of saline 25 mg/kg/week or mcr84, or daily oral gavage of 50 mg/kg/week sunitinib. Therapy continued for a total of eight weeks, at which time all animals were sacrificed. Tumor burden, as assessed by final pancreas weight at sacrifice, was reduced in mcr84-treated animals as compared to control- and sunitinib-treated animals, although this trend failed to reach significance (Figure S2C). However, treatment with mcr84 or sunitinib resulted in decreased tumor MVD (Figure S2D, p<0.01 or p<0.05, respectively). The effects of r84 in PDAC and its possible therapeutic benefit in animal models of PDAC are active areas of research in our lab. Tail cuff blood pressure measurements were gathered during weeks two and seven of therapy. At week two, animals in the mcr84 and sunitinib groups displayed elevated systolic blood pressure as compared to control-treated animals (Figure 5C, left panel, p<0.001). However, at week seven neither mcr84- nor sunitinib-treated animals displayed elevated systolic blood pressures as compared to control-treated animals although blood pressures were significantly higher in sunitinib-treated animals than in those receiving mcr84 (Figure 5C, right panel, p<0.05). Thus in this model, inhibiting VEGFR2 with mcr84 or multiple receptor tyrosine kinases including both VEGFR1 and VEGFR2 with sunitinib increased systolic blood pressure after acute but not chronic therapy. During week six of therapy, metabolic cages were used to collect urine samples from all mice, which were subsequently analyzed for urine protein (Upro) and creatine levels by UT Southwestern Medical Center's mouse metabolic phenotyping core. The Upro∶creatine ratio did not differ between the three treatment groups, suggesting that long term treatment with mcr84 and sunitinib does not induce kidney damage (Figure 5D). Similar to the initial toxicity study in NOD/SCID mice, blood was collected from all animals at sacrifice for analysis by the mouse metabolic phenotyping core, which again yielded no significant changes in any of the 18 tested analytes between mcr84-, sunitinib-, or control-treated animals (Table S2). Therefore, in a spontaneous tumor model in immunocompetent animals, chronic treatment with mcr84 failed to produce observable, lasting toxicity.

## Discussion

Angiogenesis is a crucial process during embryonic development and normal physiology, and during tumor development, growth, and progression [2]. Anti-angiogenic therapy therefore presents an exciting and rational approach for tumor therapy. However, the clinical efficacy of anti-angiogenic therapies have been mostly disappointing, with modest increases in patient overall survival [27]. Therefore, there is still much to learn about angiogenic signaling and angiogenesis dependence within tumors, which can be aided through the development and use of new investigative tools.

Here we describe r84, a fully human monoclonal antibody specific for VEGF, a key mediator of angiogenesis. r84 binds to human and mouse VEGF-A, but not other VEGF family members (VEGF-B, -C, -D, PlGF), and specifically blocks subsequent binding of VEGF to VEGFR2, leaving intact VEGF∶VEGFR1 interaction. Through its unique VEGF binding properties, r84 blocked VEGFR2-mediated endothelial cell migration and signaling. *In vivo*, r84 controlled tumor growth in NOD/SCID mice similarly to bevacizumab. r84-treated tumors had reduced MVD, VEGFR2 expression, and LVD as compared to control-treated tumors, and showed a trend towards increased pericyte-associated blood vessels. Importantly, chronic exposure to r84 in tumor bearing and non-tumor bearing NOD/SCID mice and in a spontaneous, immunocompetent model of pancreatic cancer did not induce toxicity.

The discriminating specificity of r84 in that it recognizes one ligand (VEGF) and inhibits binding only to VEGFR2 establishes r84 as a beneficial tool for elucidating VEGFR1 signaling pathways and functional contributions of VEGFR1 and VEGFR2 *in vitro* and *in vivo*. r84 binds both human and mouse VEGF (Figure 1A, B), and a mouse chimeric version of r84 (mcr84) has been developed, thereby obviating the need for complex mouse model systems genetically engineered to express human VEGF [12] to study contributions of host- and tumor-derived VEGF in human xenograft or syngeneic tumor models. Previous work has directly compared the efficacy of r84 with other anti-angiogenic agents in established human tumor xenografts and syngeneic tumor models [20]–[21]. In these studies, r84 has been shown to be more effective than bevacizumab, sunitinib, an anti-VEGFR2 antibody (RAFL-2), and a peptoid against VEGFR1 and VEGFR2 (GU81) in controlling tumor growth and infiltration of immune suppressor cell populations [19], [21]. Functionally, r84 inhibits VEGFR2 activity by specifically blocking only VEGF. This distinguishes this r84 from anti-VEGFR2 antibodies such as DC101 that block the activity of all VEGFR2 ligands [28]. The importance of r84's specificity is best observed through direct comparisons where r84 has been shown to outperform less specific anti-VEGFR2 strategies [21]. The present study supports the previous investigations, highlighting that selective inhibition of VEGFR2 with r84 can delay tumor take and control tumor growth similar to blockade of both VEGFR1 and VEGFR2 (Figure 3), bringing to question the function of VEGFR1 in tumor angiogenesis and in physiological homeostasis. A caveat to the specificity of r84 is that we have been unable to determine conclusively the effect of r84 on VEGF binding to neuropilin-1 or -2, which might impact the biological effect of r84.

Although the function and signaling pathways of VEGFR1 remain elusive, there is data supporting the concept that VEGFR1 is a negative regulator of VEGFR2 signaling. VEGFR1 deficient mice die *in utero* due to an over abundance of endothelial cells [29]–[30], whereas mice expressing only the extracellular domain of VEGFR1 are viable [31]. These studies established that VEGFR1 does not need to signal through its cytoplasmic domain and functions during development as a decoy receptor for VEGF, sequestering the ligand and regulating VEGFR2-mediated angiogenesis. Roberts *et al.*, [32] demonstrated that the VEGFR1 mutant phenotype in embryonic stem cell-derived blood vessels could be rescued by incubation with small molecule inhibitors of VEGFR2. These data further supports that VEGFR1 controls blood vessel development by negatively regulating VEGFR2 signaling. In addition, work by Nozaki *et al.*, [33] demonstrated that VEGF binding to VEGFR1 induced the activity of SHP-1 phosphatase that in turn reduced levels of VEGFR2 phosphorylation. Therefore, active VEGF binding and signaling through VEGFR1 could potentially negatively regulate tumor angiogenesis, an interesting concept that warrants further investigation. Hypertension is likely caused by decreased levels of nitric oxide (NO) resulting from blockade of VEGF signaling through VEGFR2 and VEGFR1 by current anti-angiogenic strategies. VEGF activation of VEGFR1 has been demonstrated to induce NO production [34]–[35]. Therefore, it is possible that hypertension may be reduced or eliminated following r84 therapy.

Additionally, studies have demonstrated the importance of VEGFR1 function in tumor cell survival. Neutralizing antibodies against VEGFR1 [36]–[37] and PlGF [38], a VEGFR1 specific ligand, have successfully controlled tumor growth in preclinical models. Adding to the complexity of this pathway, PlGF over expression has also been shown to inhibit tumor growth and angiogenesis through increased levels of functionally inactive VEGF∶PlGF heterodimers [39]–[40]. Further, Bais et al. recently demonstrated that although anti-PlGF antibodies were able to inhibit wound healing and cancer cell extravasation, these antibodies only inhibited tumor growth in tumors that over expressed VEGFR1 [41]. These papers question the importance of directly blocking PlGF or VEGFR1 therapeutically and highlight the potential benefit of anti-angiogenic agents such as r84 that allow for PlGF and VEGFR1 interactions. VEGFR1 has also been linked to tumor metastasis [42]–[43]. However, selective blockade of VEGFR2 in our models was sufficient to control tumor growth as compared to simultaneous inhibition of VEGFR1 and VEGFR2 (Figure 3A–D). Increased metastasis was not observed from tumor xenografts treated with r84 or the phenotypic precursor of r84, 2C3, in subcutaneous or orthotopic models [3], [20]. Nevertheless, the effects of anti-angiogenic therapy on tumor progression and metastasis are still being elucidated [44]–[45] and could benefit from selective tools, such as r84, to delineate important pathways and mechanisms of action in these processes.

In the present study, delayed tumor take through selective inhibition of VEGFR2 with r84 was associated with several histological changes. r84 reduced tumor MVD similar to bevacizumab treatment (Figure 4A). Consistent with the concept of anti-angiogenic therapies functioning by pruning nascent tumor vasculature, we observed a trend of increased pericyte association with endothelial cells in r84 and bevacizumab treated animals, though this only reached statistical significance in the H460 model (Figure 4A). NSCLC tumors treated with r84 and bevacizumab showed a reduction in VEGFR2 staining (Figure 4B), with the exception of A549 tumors where bevacizumab had no effect, suggesting specific inhibition of VEGF∶VEGFR2 binding by r84 can down regulate receptor expression. As VEGFR2 is considered the predominant angiogenic signaling receptor, decreasing its expression within tumors could promote the anti-angiogenic effects of r84. Tumor LVD was also decreased in mice treated with r84 and bevacizumab, with the exception of A549 tumors where bevacizumab had no effect. In several tumor types, including lung cancer, lymphatic vasculature participates in tumor metastasis [46]. Although predominately mediated by VEGF-C and -D interaction with VEGFR3, recent data demonstrated elevated expression of tumor-derived VEGF-A contributes to pathological lymphangiogenesis [47]–[48]. In a corneal injury model, Cursiefen *et al.*, [47] demonstrated that elevated levels of VEGF-A recruits macrophages and inflammatory cells secreting VEGF-C and -D to the site of injury, thereby inducing lymphangiogenesis. This mechanism may explain the decrease in LVD seen in treated tumors in our studies. Therefore, reduced LVD observed with r84 and bevacizumab therapy is perhaps mechanistically similar to the reduction in LVD observed in 2C3-treated breast cancer xenografts, which correlated with a VEGFR2-mediated down regulation of VEGFR3 in lymphatic endothelial cells and a decrease in Ang-2 expression in endothelial cells and tumor cells [49].

Extended therapy with r84 in tumor bearing and non-tumor bearing mice did not induce toxicity, as measured by weight maintenance, blood pressure levels, proteinuria analysis, and preservation of renal, hepatic, and pancreatic structure and function. Previous studies assessing the safety of anti-VEGF antibodies, including bevacizumab, demonstrated increased hepatic and renal damage with antibodies of increasing affinity to VEGF. Hepatic and renal toxicity produced elevated serum levels of ALT, AST, and BUN as well as glomerulosclerosis and loss of structural integrity seen by H&E staining [12]. These toxicity-inducing antibodies were first characterized in 2006 as cross-reactive antibodies that recognized human and mouse VEGF and highlighted the importance of blocking stromal-derived VEGF in some tumor models [50]. Our current work with r84 in the A549 xenograft model (Figure 3C–D) highlights the importance of host VEGF in the progression of some tumors. However, in our studies, long-term therapy with r84 does not induce the renal or hepatic toxicities (Figure 5, Table S1). This separates r84 from previously developed cross-reactive antibodies as a unique therapeutic tool with the potential to answer key questions on the function of stromal VEGF in tumor progression and the importance of VEGFR1 activity in avoiding anti-VEGF induced toxicity. The endocrine pancreas is especially sensitive to VEGF inhibition [9], [51]. However, extended therapy with r84 did not result in changes in pancreatic islet structure or function (Figure S3A-C). In an immunocompetent model of spontaneous pancreatic cancer, extended therapy with mcr84 did not induce renal or hepatic toxicities as indicated by urine analysis and serum metabolic markers (Figure 5D, Table S2) and acute increases in systolic blood pressure were resolved over time without cessation of therapy (Figure 5C). Thus, we conclude that r84 and mcr84 do not induce significant toxicities in mice perhaps due to the lower affinity of r84 for VEGF as compared to other anti-VEGF antibodies or from a protective function of VEGFR1. Overall, the *in vitro* and *in vivo* characteristics of r84 establish this antibody as an important tool to further elucidate the importance of VEGF signaling through VEGFR1 and VEGFR2 within tumors and during normal physiology and as a potential adjuvant therapy. At the present time the production of clinical grade r84 is being evaluated and we anticipate that initial safety trials in humans will begin in the near future.

## Supporting Information

Table S1 Extended r84 therapy does not induce significant changes in blood serum chemistry. NOD/SCID mice bearing subcutaneous PANC-1 tumors received long-term 12-week therapy with 50 mg/kg/week r84 or a control IgG. Blood chemistry analysis of serum samples collected from mice at sacrifice indicated that extended r84 treatment does not induce changes in serum levels of 20 different markers, as compared to control-treated (Ctrl) or Naïve animals.
(0.03 MB DOC)

Table S2 Extended mcr84 therapy does not induce significant changes in blood serum chemistry. Immunocompetent mice heterozygous for a spontaneous model of pancreatic cancer received extended 8-week therapy with saline, 25 mg/kg/week mouse chimeric r84 (mcr84), or 50 mg/kg/week sunitinib. Blood chemistry analysis of serum samples collected from mice at sacrifice indicated that extended mcr84 and sunitinib treatment does not induce changes in serum levels of 18 different markers, as compared to saline-treated animals in this model.
(0.03 MB DOC)

Figure S1 r84 reduces mouse VEGF-induced endothelial cell migration and signaling *in vitro*. A, A modified Boyden chamber migration assay was used to assess the effect of r84, bevacizumab (bev) on mouse VEGF-induced endothelial cell (EC) migration. 20,000 PAE-KDR cells were plated on 8.0 µm cell culture inserts and allowed to migrate overnight towards SFM or mouse VEGF (100 ng/mL)+/−500-fold molar excess antibody (bev, r84, control IgG). Only r84 blocks mouse VEGF-induced migration of VEGFR2-expressing PAE-KDR ECs. B, Western blots of mouse VEGF-induced signaling in PAE-KDR lysates following stimulation of cells with 50 ng/mL mouse VEGF+/−500-fold molar excess antibody (bev, r84, control IgG). Only r84 blocks p-VEGFR2 and downstream phosphorylation (PLC-γ, ERK1/2) in mouse VEGF-stimulated cells. ***p<0.001, statistical differences in A compared to mouse VEGF alone, unless otherwise indicated.
(0.68 MB TIF)

Figure S2 Efficacy of long-term anti-VEGF therapy. r84 and mcr84 were able to control tumor growth in two extended therapy models. A–B, NOD/SCID mice bearing subcutaneous PANC-1 tumors received long-term 12-week therapy with 50 mg/kg/week r84 or a control IgG. r84 therapy significantly controls tumor growth and final tumor weight compared to control IgG (A, *p<0.05). B, r84 significantly decreases PANC-1 tumor microvessel density as compared to control IgG (Ctrl) treatment as shown by endomucin staining (***p<0.001). C, Immunocompetent mice heterozygous for a spontaneous model of pancreatic cancer received extended 8-week therapy with saline, 25 mg/kg/week mouse chimeric r84 (mcr84), or 50 mg/kg/week sunitinib. There was a trend towards a decrease in final pancreas weight at time of sacrifice in mcr84-treated animals as compared to control, although this decrease failed to reach statistical significance.
(3.99 MB TIF)

Figure S3 Immunohistochemical analysis of r84 efficacy and toxicity profile following long-term therapy. NOD/SCID mice bearing subcutaneous PANC-1 tumors received long-term 12-week therapy with 50 mg/kg/week r84 or a control IgG. A, Long-term r84 therapy in TB or NTB animals did not change pancreatic islet vessel density (endomucin, green) or pericyte distribution (NG2, red) as compared to age-matched Naïve animals (**p<0.01). Blood chemistry analysis of serum samples collected from mice at sacrifice revealed no change in glucose levels between groups (B). TB or NTB animals receiving long-term antibody therapy with r84 or a control IgG and Naïve animals showed no difference in insulin staining intensities (green) within pancreatic islets (C).
(7.39 MB TIF)
