# Supplementary material for: r84, a Novel Therapeutic Antibody against Mouse and Human VEGF with Potent Anti-Tumor Activity and Limited Toxicity Induction
Source: PLoS One. 2010 Aug 6;5(8):e12031. doi: 10.1371/journal.pone.0012031 (PMC2917360; doi:10.1371/journal.pone.0012031)
Supplement: Table S1 — Extended r84 therapy does not induce significant changes in blood serum chemistry. NOD/SCID mice bearing subcutaneous PANC-1 tumors received long-term 12-week therapy with 50 mg/kg/week r84 or a control IgG. Blood chemistry analysis of serum samples collected from mice at sacrifice indicated that extended r84 treatment does not induce changes in serum levels of 20 different markers, as compared to control-treated (Ctrl) or Naïve animals. (0.03 MB DOC) [file pone.0012031.s001.doc]

**Supplementary Table 1.**

**Supplementary Table 1. Extended r84 therapy does not induce significant changes in blood serum chemistry.** NOD/SCID mice bearing subcutaneous PANC-1 tumors received long-term 12-week therapy with 50 mg/kg/week r84 or a control IgG. Blood chemistry analysis of serum samples collected from mice at sacrifice indicated that extended r84 treatment does not induce changes in serum levels of 20 different markers, as compared to control-treated (Ctrl) or Naïve animals.
