# Supplementary material for: r84, a Novel Therapeutic Antibody against Mouse and Human VEGF with Potent Anti-Tumor Activity and Limited Toxicity Induction
Source: PLoS One. 2010 Aug 6;5(8):e12031. doi: 10.1371/journal.pone.0012031 (PMC2917360; doi:10.1371/journal.pone.0012031)
Supplement: Table S2 — Extended mcr84 therapy does not induce significant changes in blood serum chemistry. Immunocompetent mice heterozygous for a spontaneous model of pancreatic cancer received extended 8-week therapy with saline, 25 mg/kg/week mouse chimeric r84 (mcr84), or 50 mg/kg/week sunitinib. Blood chemistry analysis of serum samples collected from mice at sacrifice indicated that extended mcr84 and sunitinib treatment does not induce changes in serum levels of 18 different markers, as compared to saline-treated animals in this model. (0.03 MB DOC) [file pone.0012031.s002.doc]

**Supplementary Table 2.**

**Supplementary Table 2. Extended mcr84 therapy does not induce significant changes in blood serum chemistry.** Immunocompetent mice heterozygous for a spontaneous model of pancreatic cancer received extended 8-week therapy with saline, 25 mg/kg/week mouse chimeric r84 (mcr84), or 50 mg/kg/week sunitinib. Blood chemistry analysis of serum samples collected from mice at sacrifice indicated that extended mcr84 and sunitinib treatment does not induce changes in serum levels of 18 different markers, as compared to saline-treated animals in this model.
