# Supplementary figures and images for: r84, a Novel Therapeutic Antibody against Mouse and Human VEGF with Potent Anti-Tumor Activity and Limited Toxicity Induction
Source: PLoS One. 2010 Aug 6;5(8):e12031. doi: 10.1371/journal.pone.0012031 (PMC2917360; doi:10.1371/journal.pone.0012031)

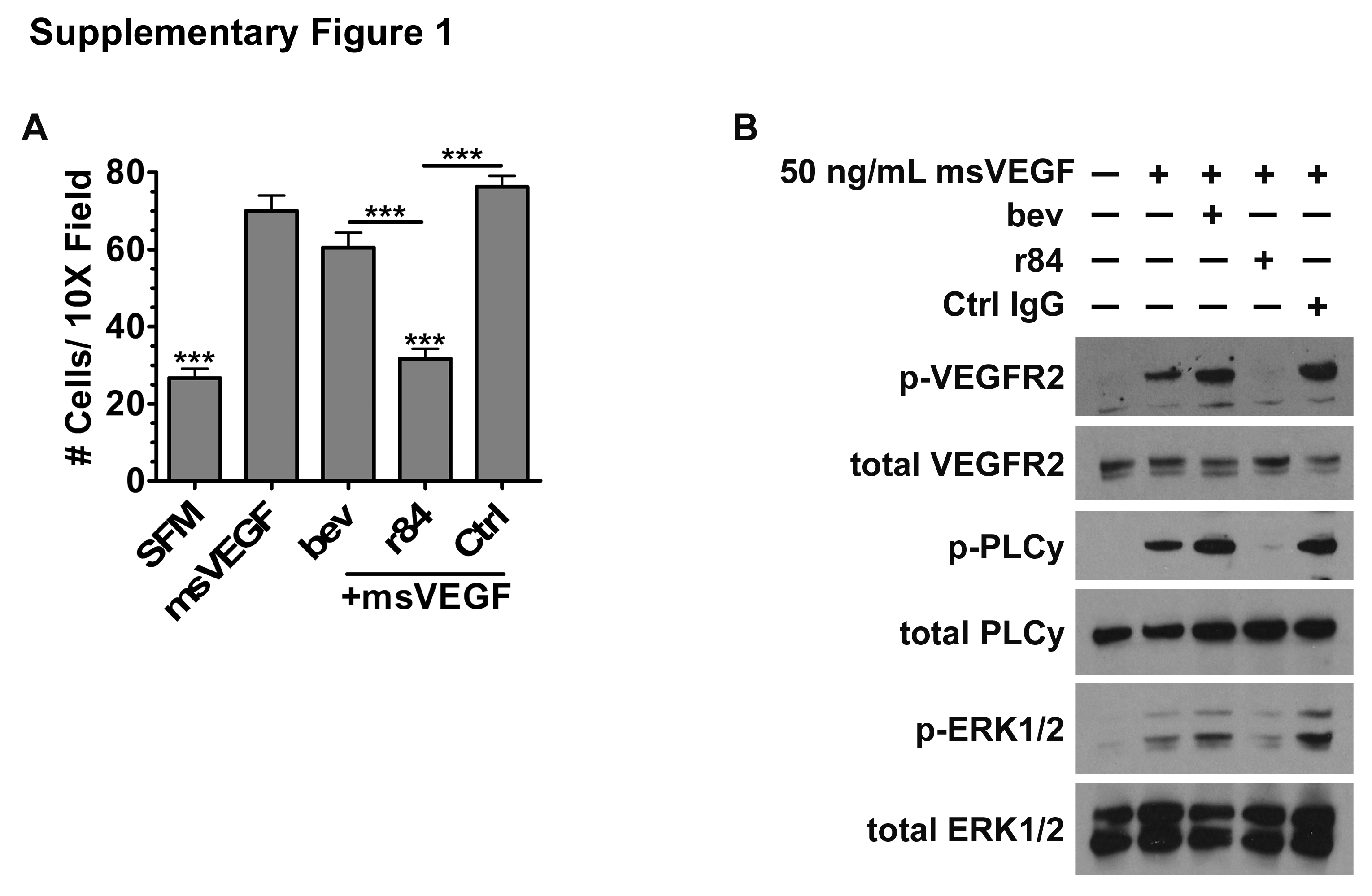

Supplement: Figure S1 — r84 reduces mouse VEGF-induced endothelial cell migration and signaling in vitro. A, A modified Boyden chamber migration assay was used to assess the effect of r84, bevacizumab (bev) on mouse VEGF-induced endothelial cell (EC) migration. 20,000 PAE-KDR cells were plated on 8.0 µm cell culture inserts and allowed to migrate overnight towards SFM or mouse VEGF (100 ng/mL)+/−500-fold molar excess antibody (bev, r84, control IgG). Only r84 blocks mouse VEGF-induced migration of VEGFR2-expressing PAE-KDR ECs. B, Western blots of mouse VEGF-induced signaling in PAE-KDR lysates following stimulation of cells with 50 ng/mL mouse VEGF+/−500-fold molar excess antibody (bev, r84, control IgG). Only r84 blocks p-VEGFR2 and downstream phosphorylation (PLC-γ, ERK1/2) in mouse VEGF-stimulated cells. ***p<0.001, statistical differences in A compared to mouse VEGF alone, unless otherwise indicated. (0.68 MB TIF) [file pone.0012031.s003.tif]

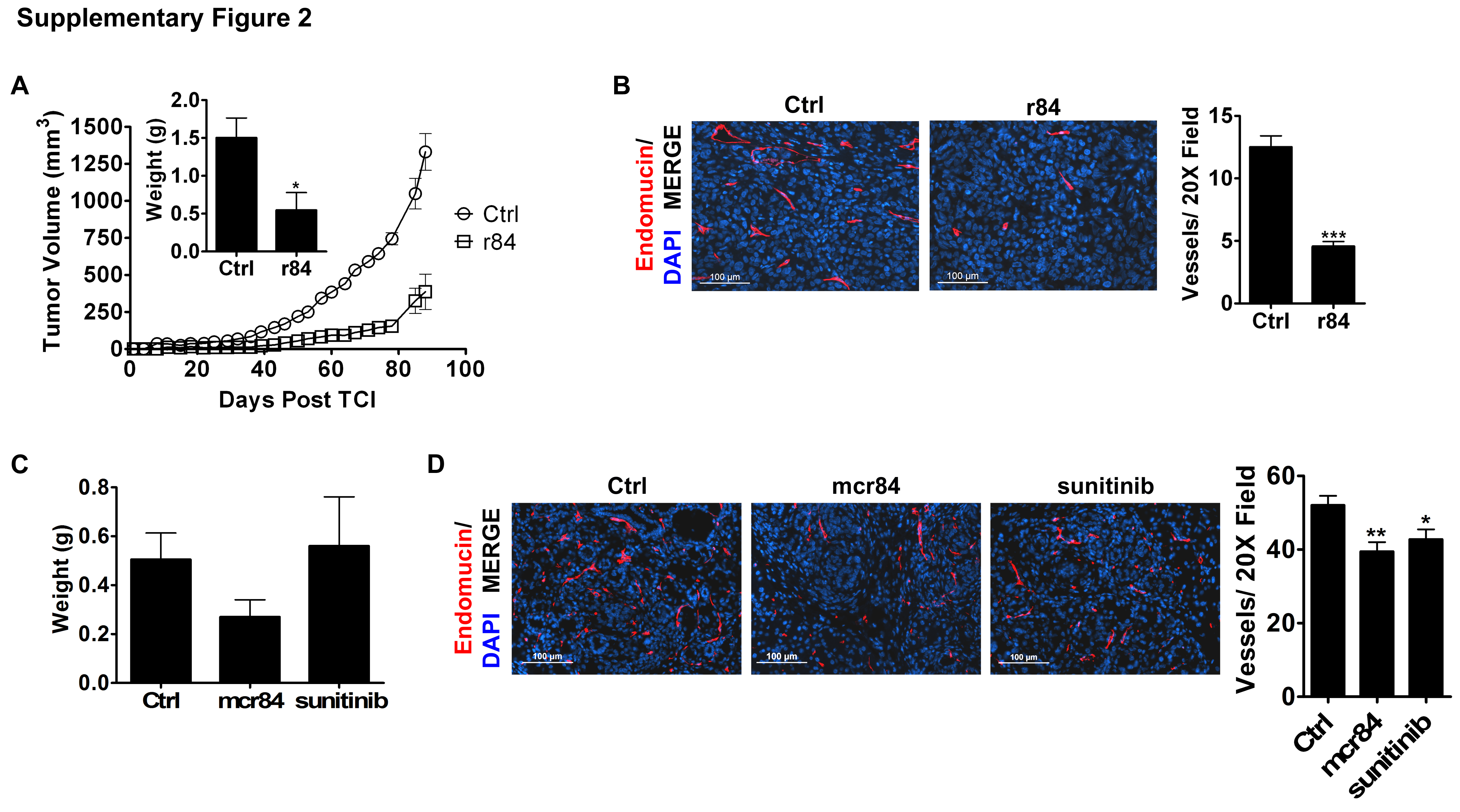

Supplement: Figure S2 — Efficacy of long-term anti-VEGF therapy. r84 and mcr84 were able to control tumor growth in two extended therapy models. A–B, NOD/SCID mice bearing subcutaneous PANC-1 tumors received long-term 12-week therapy with 50 mg/kg/week r84 or a control IgG. r84 therapy significantly controls tumor growth and final tumor weight compared to control IgG (A, *p<0.05). B, r84 significantly decreases PANC-1 tumor microvessel density as compared to control IgG (Ctrl) treatment as shown by endomucin staining (***p<0.001). C, Immunocompetent mice heterozygous for a spontaneous model of pancreatic cancer received extended 8-week therapy with saline, 25 mg/kg/week mouse chimeric r84 (mcr84), or 50 mg/kg/week sunitinib. There was a trend towards a decrease in final pancreas weight at time of sacrifice in mcr84-treated animals as compared to control, although this decrease failed to reach statistical significance. (3.99 MB TIF) [file pone.0012031.s004.tif]

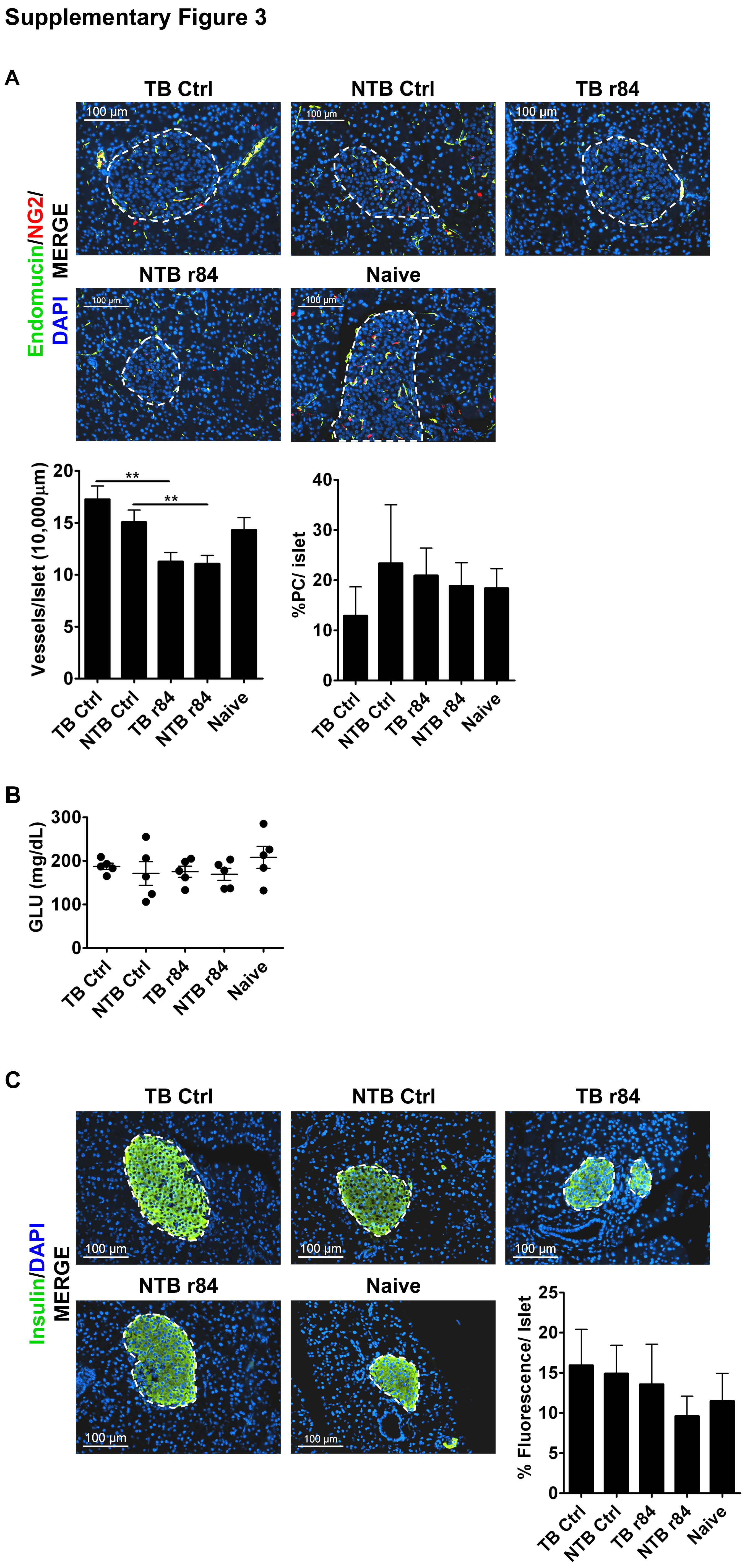

Supplement: Figure S3 — Immunohistochemical analysis of r84 efficacy and toxicity profile following long-term therapy. NOD/SCID mice bearing subcutaneous PANC-1 tumors received long-term 12-week therapy with 50 mg/kg/week r84 or a control IgG. A, Long-term r84 therapy in TB or NTB animals did not change pancreatic islet vessel density (endomucin, green) or pericyte distribution (NG2, red) as compared to age-matched Naïve animals (**p<0.01). Blood chemistry analysis of serum samples collected from mice at sacrifice revealed no change in glucose levels between groups (B). TB or NTB animals receiving long-term antibody therapy with r84 or a control IgG and Naïve animals showed no difference in insulin staining intensities (green) within pancreatic islets (C). (7.39 MB TIF) [file pone.0012031.s005.tif]
